# A Hepatitis C Educational Needs Assessment of Canadian Healthcare Providers

**DOI:** 10.1155/2017/5324290

**Published:** 2017-03-15

**Authors:** Reza Naghdi, Karen Seto, Carolyn Klassen, Didi Emokpare, Brian Conway, Melissa Kelley, Eric Yoshida, Hemant A. Shah

**Affiliations:** ^1^Toronto Centre for Liver Disease, University Health Network, University of Toronto, Toronto, ON, Canada; ^2^Canadian Liver Foundation, National Office, Markham, ON, Canada; ^3^Canadian Association of Hepatology Nurses, Ottawa, ON, Canada; ^4^Outpost Healthcare, Regina, SK, Canada; ^5^Vancouver Infectious Disease Centre, Vancouver, BC, Canada; ^6^Faculty of Medicine, Memorial University, St. John's, NL, Canada; ^7^Division of Gastroenterology, Vancouver General Hospital and University of British Columbia, Vancouver, BC, Canada

## Abstract

*Background and Aim*. Despite advances in the treatment of chronic hepatitis C infection (CHC), it remains a major public health problem in Canada and globally. The knowledge of healthcare providers (HCPs) is critical to improve the care of CHC in Canada. To assess the current knowledge and educational needs of healthcare providers (HCPs) in the area of CHC management a national online survey was conducted.* Method*. An interprofessional steering committee designed a 29-question survey distributed through various direct and electronic routes. The survey assessed several domains (e.g., participant and practice demographics, access to resources, knowledge of new treatments, and educational preferences).* Results*. A total of 163 HCPs responded to the survey. All hepatologists and 8% of primary care providers (PCPs) reported involvement in treatment of CHC. Physicians most frequently screened patients who had abnormal liver enzymes, while nurses tended to screen based on lifestyle factors. More than 70% of PCPs were not aware of new medications and their mechanisms.* Conclusion*. Overall, the needs assessment demonstrated that there was a need for further education, particularly for primary care physicians, to maximize the role that they can play in screening, testing, and treatment of hepatitis C in Canada.

## 1. Introduction

Chronic hepatitis C (CHC) is one of the leading causes of liver disease, including cirrhosis and hepatocellular carcinoma. Between 60 and 85% of individuals exposed to the hepatitis C virus (HCV) develop a chronic infection [1].

In the past, treatment for CHC was recommended for patients with increased risk of cirrhosis [1] and others might undergo periodic monitoring rather than treatment. Because of the limited treatment options, complexity of treatment, and reimbursement challenges, many physicians have not administered treatment of CHC in their practices.

Recent advances in CHC treatment have made it the first chronic viral infection to be reliably curable through pharmacotherapy, with newer antiviral medications demonstrating cure rates approaching 100% [2]. These new antiviral agents are now available to Canadian patients and their healthcare providers (HCPs). However, with rapid advances in HCV science including new treatment modalities, ensuring that the treatments are reaching individuals who would most benefit presents a challenging educational problem [2]. To bridge this gap, further education for a wide variety of HCPs is required. This education should include how to screen for patients with chronic infection, the selection of patients, and the treatment itself. In order to develop optimal education programs, a national needs assessment was conducted.

## 2. Methodology

### 2.1. Study Design and Content

The needs assessment was conducted by the Canadian Liver Foundation (CLF) and the Canadian Association for the Study of the Liver (CASL), in partnership with the Canadian Association of Hepatology Nurses (CAHN). The intent of the assessment was to determine the educational needs of Canadian healthcare professionals in the area of hepatitis C treatment, particularly in light of the availability of new antiviral agents. The initiative was guided through a steering committee composed of individuals with expertise in the field, representing the following healthcare specialties: hepatology, gastroenterology, infectious disease, hepatology nursing, and primary care.

The survey content was developed and then circulated through the steering committee, CLF, CASL, and CAHN. The survey consisted of 29 questions collecting participant and practice demographics, access to resources, screening habits, communication, knowledge of new treatments, and educational preferences Appendix. The survey commenced on November 14, 2014, and concluded on April 29, 2015.

### 2.2. Study Population

Invitations to participate were distributed through various means, including direct peer-to-peer interactions by steering committee members, and by distribution to members of the CLF, members of CASL, and members of CAHN. Invitations were also delivered by facsimile and email to a targeted list of approximately 3500 individuals in the fields of hepatology, gastroenterology, pediatric gastroenterology, tropical travel medicine, infectious disease, sexually transmitted disease, communicable disease, hospitalist, native medicine, prison/detention medicine, internal medicine, general care, and family medicine physicians.

### 2.3. Data Collection and Analysis

Partially completed surveys (i.e., those that did not have all questions completed) were accepted, with analysis based on the number of respondents for each question. Data analysis was simple summary statistics and included analysis of all participants (aggregated), as well as participants categorized by their declared medical role/specialization. For a series of questions specific to participant comfort for various activities, the average level of comfort was calculated. Each level of comfort ranging from very uncomfortable to very comfortable was assigned a value from 1 to 5, respectively. The average of the comfort level value was then calculated to yield a value from 1 to 5, with 1 representing very uncomfortable and 5 representing very comfortable.

### 2.4. Ethics

The final survey was IRB approved (Veritas IRB) with distribution and results collected online through https://www.surveymonkey.com/.

## 3. Results 

### 3.1. General Characteristics

163 complete or partial survey responses were received. Of 163 participants who began the survey, 153 participants answered all questions and 10 surveys were incomplete. The participants were categorized into 5 groups: hepatology nurses and nurse practitioners who were grouped as nursing (*n* = 75), hepatologists (*n* = 33), gastroenterologists (*n* = 20), other specialists (*n* = 11), and primary care physicians (PCPs) (*n* = 24). The largest proportion of participants reported practicing in Ontario (44%), followed by British Columbia, Alberta, and Quebec (18%, 13%, and 12%, resp.).

### 3.2. Patient Subpopulations

The most common subpopulation identified as being seen in clinical practice were patients with cirrhosis (noted by 80% of participants). 70% of nurses reported seeing injection drug users and an additional 60% of nurses reported seeing individuals from the aboriginal population.

### 3.3. Provision of Care

All hepatologists reported involvement in the treatment of CHC, while it was reported to be 55% for gastroenterologists and 64% for other specialists. Only 8% of PCPs noted prescribing treatment for CHC as one of their aspects of care, while 92% reported a role in screening and diagnosis. In contrast, only 61% of hepatologists reported involvement in screening and diagnosis (Table 1).

**Figure 1:**
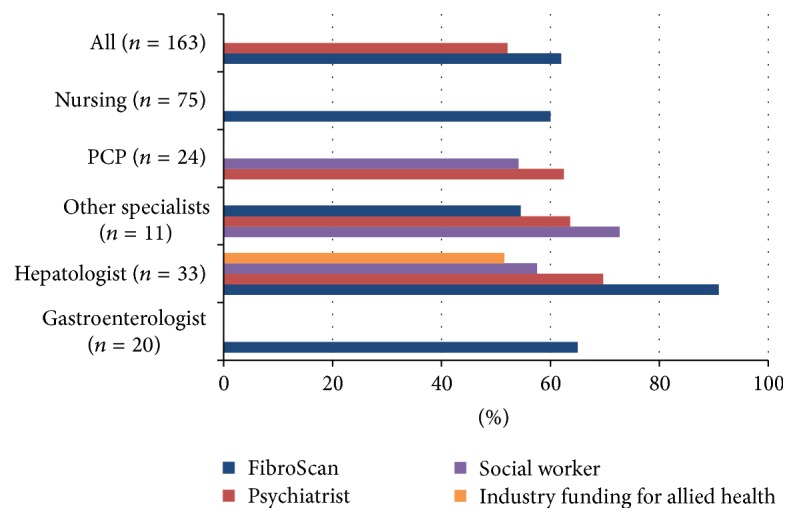
Access to HCV practice needs by specialization.

Most participants reported inadequate access to both telehealth and government funding for allied health (only 29% and 31% of participants had access, resp.). There were only four resources that were available to more than half the participants in any specialty: FibroScan® (transient elastography), social workers, psychiatry consultation, and industry funding for allied health (Figure 1).

### 3.4. Screening

**Figure 2:**
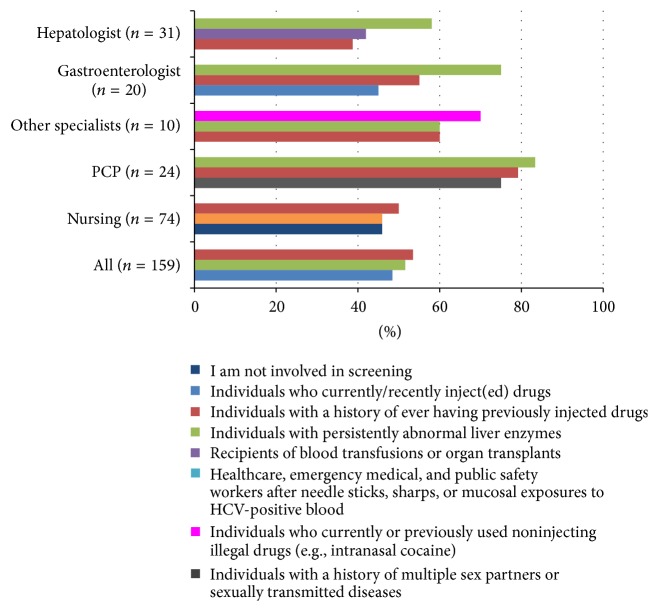
Participants selection factors for screening patients for hepatitis C.

When selecting patients to screen for CHC, physicians most frequently tested patients with abnormal liver enzymes, while nurses tended to select patients based on lifestyle risk factors (injection drug use, body piercing, etc.). However, nurses reported less involvement with screening in general. Notably, PCPs tended to screen based on a wide variety of factors (i.e., from abnormal liver enzymes to medical and lifestyle history) (Figure 2).

The majority of the participants were either unsure or did not think that current screening guidelines would be able to identify all persons infected with hepatitis C. Hepatologists (84% certain, 6% unsure) had the highest level of certainty that current screening guidelines would not identify all infected persons. Nurses were the most likely to think that screening could identify all infected persons (24%).

### 3.5. Recommending and Initiating Treatment

There was a general decrease in the comfort level of recommending treatment as patients were described as being more advanced, from mild fibrosis to decompensated patients. Overall, 74% of participants were comfortable or very comfortable recommending therapy for asymptomatic patients, 75% for symptomatic patients, and 58% for decompensated patients. PCPs were generally very uncomfortable with the role of identifying and recommending treatment (58% to 75% reported discomfort for the different patient types).

Among PCPs, 78% expressed discomfort with initiating CHC therapy, while 71% of other participants felt comfortable with initiating treatment. Highest comfort levels were found in hepatologists (with average comfort level of 4.5 out of 5). Similar results were obtained on participant comfort for switching patients from one therapy to another because of poor efficacy; 70% of PCPs felt discomfort compared with 55% of participants expressing comfort for switching. Participants expressed greater comfort in monitoring patients' current therapy with an average comfort level of 4.10 out of 5 for all participants, but again lower for PCPs with an average comfort of 2.83.

### 3.6. Communication

**Figure 3:**
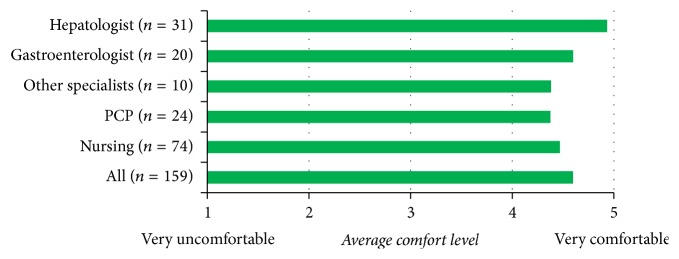
Level of comfort of the participants in explaining the necessity of hepatitis C testing.

A large majority of participants (78% to 96%) were either comfortable or very comfortable in explaining the necessity of hepatitis C testing (Figure 3). A lower proportion of nursing participants were either comfortable or very comfortable explaining hepatitis C testing, though the average comfort level in this group was higher than PCPs.

Specialty and primary care providers both had lower levels of comfort educating patients about overcoming stigma than about emerging treatments.

### 3.7. Knowledge of New Treatments

**Figure 4:**
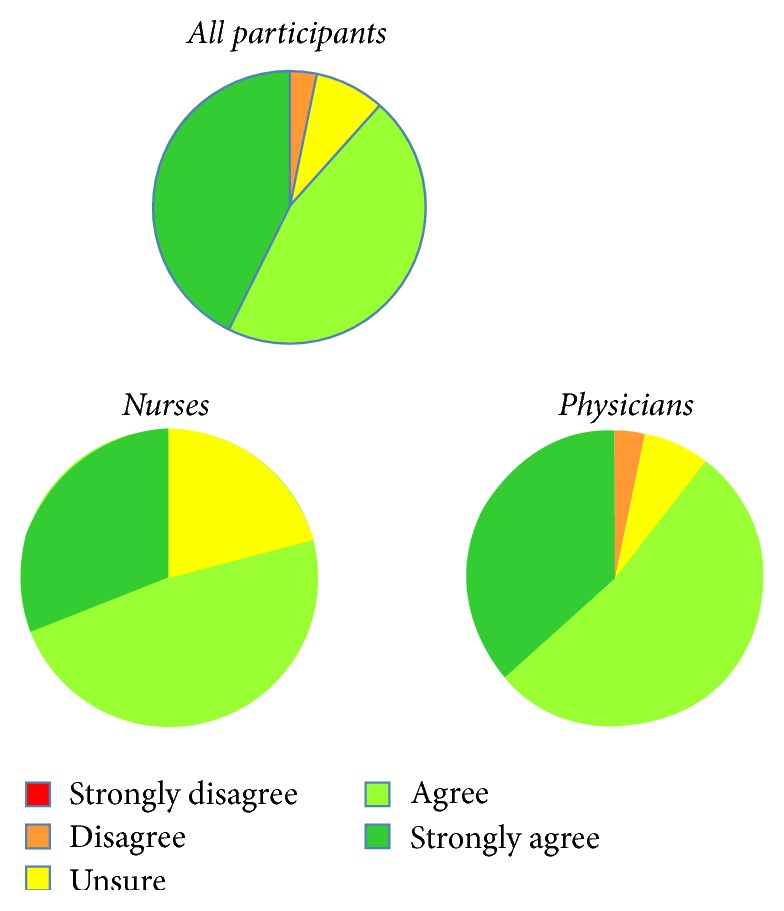
Agreement of the participants on curability of hepatitis C within the next five years.

Overall, a large majority of participants agreed that hepatitis C would be a curable disease for almost all patients within five years; 21% of nurses were unsure of the statement, while 11% of physicians were unsure or disagreed with the statement (Figure 4). Seven of 10 PCPs were unsure or were not aware of new direct-acting antiviral medications (DAAs), while 74% were not sure of the mechanisms of action. This contrasts with 81% of the other participants who agreed they were aware of new DAAs and 73% who agreed that they understood their mechanisms of action.

While 74% of participants agreed that they were aware of the medications, only 66% agreed that they were able to keep themselves up to date on recent clinical trial results. PCPs reported the lowest ability to keep up to date in this regard (78% unsure or disagreeing).

Overall, 63% of participants agreed that they were aware of current coverage (either formulary or private) for hepatitis C drugs. While PCPs had the lowest awareness at 22%, hepatologists reported the strongest awareness with 74% agreeing that they were aware.

### 3.8. Education

**Figure 5:**
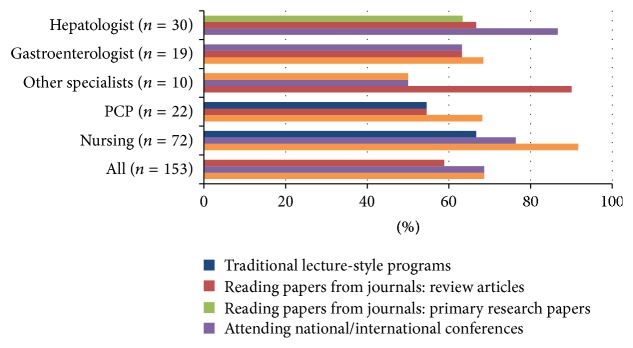
Preferred format of learning hepatitis C by specialization.

PCPs, gastroenterologists and nurses most frequently cited local conferences as a preferred format for learning; however only 1 in 5 hepatologists preferred that method. Hepatologists preferred national and international conferences (Figure 5). Participants were generally less favorable towards preceptorships (20%), online webinars (32%), or primary research papers (34%). Reading review articles was a generally accepted learning activity, with traditional lecture-style programs acceptable to the majority of participants in all groups except hepatologists. Participants (except hepatologists) were generally comfortable with the concept of conferring with HCV experts through regularly scheduled telemedicine calls.

The majority of participants reported consulting more than one set of guidelines; the most common guidelines cited were the consensus guidelines from the Canadian Association for the Study of Liver (CASL) [3] (51% of participants) and the American Association for the Study of Liver Disease (AASLD)/Infectious Disease Society of America (IDSA) guidelines [4] (47% of participants). PCPs most frequently reported other guidelines (59%) or not using any guidelines at all (45%).

## 4. Discussion

While recent advances have made CHC reliably curable, incomplete knowledge of HCPs on hepatitis C is a barrier limiting access for qualified patients who could benefit [2]. The results of this survey demonstrate various educational needs with respect to CHC among HCPs.

Before a patient with CHC can access therapy, they must be diagnosed. In Canada, underdiagnosis of CHC is a significant problem [2, 5]. The results of our study showed differences in screening criteria use between professional groups. Moreover, all 5 professional groups reported different levels of confidence in the ability of screening recommendations to find all infected patients. This demonstrates a need for improved CHC national screening guidelines and the need for increased education about them.

A significant finding of this survey was the disparate access to technologies, financial support, and interprofessional team members needed to provide CHC care especially to vulnerable populations. Government initiatives to increase treatment uptake must be accompanied by programs to scale-up access to the resources needed to achieve the best outcomes.

PCPs reported low awareness of DAAs and their mechanisms and also the lowest ability to keep up to date with new DAAs. In practice, this could translate to a lack of awareness about the highly effective nature of DAAs while having minimal side effects. This highlights the need for specific educational initiatives and designing new guidelines for the primary care audience. There were some marked differences in comfort levels in both treatment and monitoring patients. Specialists were the most comfortable with recommending and initiating treatment for CHC, while PCPs were particularly uncomfortable with those roles. This finding is consistent with other studies on PCP knowledge of new DAAs and comfort level with treatment [6]. The finding highlights the need to educate PCPs with novel and longitudinal educational models if PCPs are to become a source of increased capacity for care in the future.

There are several limitations to this study. First, the relatively small sample size limits the ability to be confident about the generalizability of results and any correlations seen in the descriptive analysis. Second, given that our participants were self-selected, selection bias is a concern. Participants with more knowledge or interest were more likely to participate in the study. Therefore, the results may overestimate the knowledge level of HCPs. Third, due to the nature of the recruitment efforts in which a unique individual may have been approached more than once, an exact survey response rate is difficult to report. Finally, this study was conducted in 2014-2015 and the therapeutic options for patients with CHC are rapidly evolving, so results relating to treatment educational needs may not apply for very long. Despite the limitations, our study did provide some important learning.

## 5. Conclusion

This study highlights several areas where HCPs perceive a high need for education including screening, patient education, and treatment. Further studies of PCPs who are the principle point of contact between the public and the healthcare system are recommended to determine the specific educational needs of this group of HCPs. Creating and continually updating the Canadian guidelines and encouraging HCPs to study and follow them with multimodal educational programs could be an appropriate solution to address the lack of knowledge about CHC and its promising new treatment.

## Figures and Tables

**Table 1:** Hepatitis C care delivered by specialization.

Which aspects of hepatitis C care are you involved in (select all that apply)?	Hepato. (*n* = 33)	Gastro. (*n* = 20)	Oth. spec. (*n* = 11)	PCP (*n* = 24)	Nursing (*n* = 75)	All (*n* = 163)
Screening/diagnosis	61%	75%	64%	92%	65%	69%
Educating/counselling diagnosed persons	82%	65%	82%	54%	93%	81%
Making treatment decisions	94%	60%	91%	21%	57%	62%
Prescribing treatment	100%	55%	64%	8%	23%	43%
Adjusting treatment	91%	50%	64%	17%	67%	62%
Dealing with adverse effects of therapy	91%	55%	82%	33%	80%	72%
Monitoring after treatment is completed	94%	65%	64%	42%	77%	73%
No response	0%	20%	0%	33%	3%	9%

## Appendix

### Survey for Online Programming

*Needs Assessment in Hepatitis C for Canadian Healthcare Professionals.* Thank you for agreeing to share your expert insights to help the* Canadian Association for the Study of the Liver (CASL)* and* Canadian Liver Foundation (CLF)*, in partnership with the* Canadian Association of Hepatology Nurses (CAHN)*, shape future education on hepatitis C screening and treatment.

You are among a select group of hepatitis C treaters that has been asked to answer an online survey assessing your perceptions of (1) knowledge of hepatitis C in Canada; (2) needs for further learning on screening for hepatitis C; and (3) needs for further learning on management of patients with hepatitis C infection.

This survey should take no more than 10 minutes of your time to complete.

Your candor is appreciated and will help us tremendously in the development of clinically applicable educational programming to serve your learning needs.

*Demographic Information*

1. Type of healthcare professional
  - Gastroenterologist
  - Hepatologist
  - Infectious disease specialist
  - Internist
  - Nurse
  - Nurse practitioner
  - Primary care physician
  - Travel medicine specialist
2. How long have you been caring for patients with hepatitis C?
  - 0 to <1 year
  - 1 to <3 years
  - 3 to <5 years
  - 5 to <10 years
  - 10 to <15 years
  - 15 years or more
3. Which of the following subpopulations of patients with hepatitis C do you see regularly in your practice (select all that apply)?
  - Individuals currently incarcerated in federal institutions
  - Individuals currently incarcerated in provincial institutions
  - Aboriginal/First Nations/Inuit individuals
  - Patients with cirrhosis
  - People who inject drugs (PWID), known use in the past 12 months
  - HIV-coinfected patients
  - Immigrants
  - Solid organ transplant recipients/bone marrow or stem cell transplant recipients
4. Which aspects of hepatitis C care are you involved in (select all that apply)?
  - Screening/diagnosis
  - Educating/counselling diagnosed persons
  - Making treatment decisions
  - Prescribing treatment
  - Adjusting treatment
  - Dealing with adverse effects of therapy
  - Monitoring after treatment is completed
5. Province/territory of practice
  - Newfoundland and Labrador
  - Nova Scotia
  - Prince Edward Island
  - New Brunswick
  - Quebec
  - Ontario
  - Manitoba
  - Saskatchewan
  - Alberta
  - British Columbia
  - Nunavut
  - Northwest Territories
  - Yukon
6. In my home province/territory, I treat patients on First Nations reserves:
  - Yes
  - No
7. In my practice, I have access to (click all that apply)
  - FibroTest,
  - FibroScan,
  - telehealth,
  - psychiatrist,
  - social worker,
  - industry funding for allied health,
  - government funding for allied health.

*Screening for Hepatitis C*

(8) Which types of patients do you currently routinely screen for hepatitis C (click all that apply)?
  - I am not involved in screening
  - Individuals who currently/recently inject(ed) drugs
  - Individuals with a history of ever having previously injected drugs
  - Individuals who have received clotting factor concentrates
  - Individuals who have been on long-term hemodialysis
  - Individuals with persistently abnormal liver enzymes
  - Individuals who have HIV infection
  - Recipients of blood transfusions or organ transplants
  - Recipients of transplanted tissue (e.g., corneal, musculoskeletal, skin, ova, sperm, solid organs, and bone marrow/stem cell transplant recipients)
  - Healthcare, emergency medical and public safety workers after needle sticks, sharps, or mucosal exposures to HCV-positive blood
  - Healthcare, emergency medical and public safety workers without known needle sticks, sharps, or mucosal exposures to HCV-positive blood
  - Children born to HCV-positive women
  - Long-term steady sex partners of HCV-positive persons
  - Household (nonsexual) contacts of HCV-positive persons
  - Individuals who currently or previously used noninjecting illegal drugs (e.g., intranasal cocaine)
  - Individuals with tattoos or body piercing
  - Individuals with a history of multiple sex partners or sexually transmitted diseases
  - Pregnant women
  - Individuals born from 1946 to 1964 (baby boomers)
  - General population
(9) Do you think current screening guidelines make it possible to identify all infected persons with hepatitis C?
  - Yes
  - No
  - Unsure

*Communication*.* Please indicate your level of comfort for the following questions (1 = very uncomfortable; 2 = uncomfortable; 3 = neither uncomfortable nor comfortable; 4 = comfortable; 5 = very comfortable):*

(10) How comfortable are you explaining to a patient why he/she should be tested for hepatitis C?
  - Very uncomfortable
  - Uncomfortable
  - Neither uncomfortable nor comfortable
  - Comfortable
  - Very comfortable
  - Not applicable to my practice
(11) How comfortable are you with communicating the diagnosis of hepatitis C to a patient who has tested positive?
  - Very uncomfortable
  - Uncomfortable
  - Neither uncomfortable nor comfortable
  - Comfortable
  - Very comfortable
  - Not applicable to my practice
(12) How comfortable are you with providing education and answering a patient's questions about the current and emerging treatment options for hepatitis C?
  - Very uncomfortable
  - Uncomfortable
  - Neither uncomfortable nor comfortable
  - Comfortable
  - Very comfortable
  - Not applicable to my practice
(13) How comfortable are you with helping a patient overcome the potential stigma associated with having a diagnosis of hepatitis C?
  - Very uncomfortable
  - Uncomfortable
  - Neither uncomfortable nor comfortable
  - Comfortable
  - Very comfortable
  - Not applicable to my practice

*Treatment of Hepatitis C*.* To what extent do you agree with the following statements?*

(14) Within the next five years, hepatitis C will be considered a curable disease for almost all patients.
  - Strongly disagree
  - Disagree
  - Unsure
  - Agree
  - Strongly agree
(15) I am fully aware of all the new direct-acting antiviral medications that have undergone or are undergoing clinical trial evaluation for the treatment of hepatitis C.
  - Strongly disagree
  - Disagree
  - Unsure
  - Agree
  - Strongly agree
(16) I understand the mechanisms of action of the various new direct-acting antiviral medications.
  - Strongly disagree
  - Disagree
  - Unsure
  - Agree
  - Strongly agree
(17) I am able to keep myself up to date on the most recent results from clinical trials of direct-acting antiviral therapies for hepatitis C.
  - Strongly disagree
  - Disagree
  - Unsure
  - Agree
  - Strongly agree
(18) I am aware of which hepatitis C drugs are covered by private insurers and provincial formularies.
  - Strongly disagree
  - Disagree
  - Unsure
  - Agree
  - Strongly agree

*Please indicate your level of comfort for the following questions (1 = very uncomfortable; 2 = uncomfortable; 3 = neither uncomfortable nor comfortable; 4 = comfortable; 5 = very comfortable):*

(19) How comfortable are you in identifying the best treatment and recommending therapy for an infected patient who is asymptomatic?
  - Very uncomfortable
  - Uncomfortable
  - Neither uncomfortable nor comfortable
  - Comfortable
  - Very comfortable
  - Not applicable to my practice
(20) How comfortable are you in identifying the best treatment and recommending therapy for an infected patient who is symptomatic?
  - Very uncomfortable
  - Uncomfortable
  - Neither uncomfortable nor comfortable
  - Comfortable
  - Very comfortable
  - Not applicable to my practice
(21) How comfortable are you in identifying the best treatment and recommending therapy for an infected patient who is decompensated (e.g., encephalopathy, varices, and ascites)?
  - Very uncomfortable
  - Uncomfortable
  - Neither uncomfortable nor comfortable
  - Comfortable
  - Very comfortable
  - Not applicable to my practice
(22) How comfortable are you with personally initiating therapy for hepatitis C in your practice?
  - Very uncomfortable
  - Uncomfortable
  - Neither uncomfortable nor comfortable
  - Comfortable
  - Very comfortable
  - Not applicable to my practice
(23) How comfortable are you with managing a patient on therapy once treatment is initiated, including monitoring and side effect management?
  - Very uncomfortable
  - Uncomfortable
  - Neither uncomfortable nor comfortable
  - Comfortable
  - Very comfortable
  - Not applicable to my practice
(24) How comfortable are you with switching a patient from one therapy to another in the event of lack of/inadequate response?
  - Very uncomfortable
  - Uncomfortable
  - Neither uncomfortable nor comfortable
  - Comfortable
  - Very comfortable
  - Not applicable to my practice

*Educational Needs*

(25) My preferred format for learning about hepatitis C is
  - traditional lecture-style programs,
  - small group sessions,
  - online modules (available for anytime access),
  - online webinars (scheduled),
  - reading papers from journals: review articles,
  - reading papers from journals: primary research papers,
  - attending national/international conferences,
  - attending local conferences,
  - preceptorships,
  - other.
(26) In some settings, practitioners are given the opportunity to confer with HCV experts by regularly scheduled telemedicine sessions. How comfortable would you be with this modality?
  - Very uncomfortable
  - Uncomfortable
  - Neither uncomfortable nor comfortable
  - Comfortable
  - Very comfortable
  - Not applicable to my practice

To what extent do you agree with the following statements?

(27) I prefer to attend educational programs sponsored by professional associations, for example, Canadian Association for the Study of the Liver (CASL), the Canadian Liver Foundation (CLF), or the Canadian Association of Hepatology Nurses (CAHN) rather than educational programs sponsored by the manufacturers of new treatments for hepatitis C.
  - Strongly disagree
  - Disagree
  - Unsure
  - Agree
  - Strongly agree
(28) Which clinical practice guidelines for hepatitis C do you use to help guide your treatment of a patient with hepatitis C?
  - Consensus guidelines from the Canadian Association for the Study of the Liver [3]
  - American (AASLD) Recommendations for Testing, Managing, and Treating Hepatitis C (2014) (online resource)
  - European (EASL) Recommendations on Treatment of Hepatitis C (2014) (online resource)
  - Other (please specify)
  - Whichever guidelines are most recent, as long as they are from a credible source
  - I do not tend to consult guidelines
(29) What would be the ideal frequency for formal updates of the Canadian guidelines for hepatitis C?
  - More frequently than twice per year
  - Twice per year
  - Annually
  - Every two years
  - Less frequently than every two years
  - As frequently as needed, as dictated by the evolving evidence base

Thank you for your interest in further contributing to our assessment. Type of healthcare professional

- Gastroenterologist
- Hepatologist
- Infectious disease specialist
- Internist
- Nurse
- Nurse practitioner
- Primary care physician
- Travel medicine specialist

Please provide your contact information below. You will be contacted after the survey closes to schedule your interview.

  Name: —
  Email: —
  Phone number: —
